# Identification and Functional Verification of MicroRNA-16 Family Targeting Intestinal Divalent Metal Transporter 1 (DMT1) *in vitro* and *in vivo*

**DOI:** 10.3389/fphys.2019.00819

**Published:** 2019-06-27

**Authors:** Shuxia Jiang, Shihui Guo, Huifang Li, Yingdong Ni, Wenqiang Ma, Ruqian Zhao

**Affiliations:** ^1^Key Laboratory of Animal Physiology and Biochemistry, College of Veterinary Medicine, Nanjing Agricultural University, Nanjing, China; ^2^MOE Joint International Research Laboratory of Animal Health and Food Safety, Nanjing Agricultural University, Nanjing, China

**Keywords:** microRNA-16 family, DMT1, regulation, intestine, functional verification

## Abstract

Divalent metal transporter 1 (DMT1) is a key transporter of iron uptake and delivering in human and animals. However, post-transcriptional regulation of DMT1 is poorly understood. In this study, bioinformatic algorithms (TargetScan, PITA, miRanda, and miRDB) were applied to predict, screen, analyze, and obtain microRNA-16 family members (miR-16, miR-195, miR-497, and miR-15b) targeting DMT1, seed sequence and their binding sites within DMT1 3′ untranslated region (3′ UTR) region. As demonstrated by dual-luciferase reporter assays, luciferase activity of DMT1 3′ UTR reporter was impaired/enhanced when microRNA-16 family member over-expression plasmid/its inhibitor was transfected to HCT116 cells. Corroboratively, co-transfection of microRNA-16 family member over-expression plasmid and DMT1 3′ UTR mutant reporter repressed the luciferase activity in HCT116 cells. In addition, over-expression microRNA-16 family member augmented its expression and diminished DMT1 protein expression in HCT116 cells. Interestingly, tail vein injection of miR-16 assay revealed reduced plasma iron levels, higher miR-16 expression, and lower DMT1 protein expression in the duodenum of mice. Taken together, we provide evidence that microRNA-16 family (miR-16, miR-195, miR-497, and miR-15b) is confirmed to repress intestinal DMT1 expression *in vitro* and *in vivo*, which will give valuable insight into post-transcriptional regulation of DMT1.

## Introduction

The divalent metal transporter 1 (DMT1), commonly abbreviated as DMT1, is generally considered to be key iron transporter for intestinal ferrous (Fe^2+^) iron uptake, and delivering iron to peripheral tissues via transferrin (Fleming et al., 1998; Gunshin et al., 2005). DMT1 plays a crucial role in iron absorption in the duodenum, where it is involved in dietary non-transferrin-bound iron uptake from the intestinal lumen (Fleming et al., 1997; Canonne-Hergaux et al., 1999). DMT1 also functions in the transferrin endosomal cycle of the erythroid precursors, hepatocytes, and other cells, where it transfers iron from the site of uptake through transferrin receptor, to cytosol, and to mitochondria for utilization (Fleming et al., 1998; Canonne-Hergaux et al., 2000). Abnormal expression of DMT1 is harmful to health. It is proposed that DMT1 mutant rodents exhibit microcytic, hypochromic anemia (Fleming et al., 1998; Canonne-Hergaux et al., 2000), whereas high-level expression of DMT1 contributes to neurodegenerative diseases (Salazar et al., 2008; Tian et al., 2018). A tight regulation of DMT1 expression is indispensable for maintenance of life in eukaryotes.

In light of pivotal role of DMT1 in iron homeostasis, tightly regulated DMT1 occurs at multiple levels, including the transcriptional (Xue et al., 2016), post-transcriptional (Gunshin et al., 2001; Galy et al., 2013), and post-translational levels (Foot et al., 2008). DMT1 is regulated transcriptionally by a transcription factor-hypoxia inducible factor 2α (HIF-2α) through binding to hypoxic response elements (HREs) and directly trans-activating its proximal promoter (Mastrogiannaki et al., 2009; Shah et al., 2009). Iron regulatory proteins (IRPs), play a stimulation role in upregulating DMT1 protein expression via binding to iron-responsive elements (IREs) localized in the 3′untranslated region (3′UTR) of DMT1 to increase its mRNA stability (Gunshin et al., 2001; Galy et al., 2013). Post-translational regulation of DMT1 is performed by Nedd4 family member WWP2 (ubiquitin-protein ligase) and Ndfip1 (Nedd4 WW domain-binding protein), which induces ubiquitin-mediated degradation via the lysosome and proteasome (Foot et al., 2008). Additionally, microRNAs are thought to be important regulators of inhibiting gene expression at the post-transcriptional levels.

MicroRNAs, are single-stranded RNAs of ∼22 nucleotides, which binds to 3′ UTR of their target mRNAs, thereby resulting in translation repression or mRNA destabilization (Bartel, 2009; Tilghman et al., 2012). Several microRNAs have been observed to regulate target genes with vital functions in iron homeostasis. The liver-specific miR-122 is known to directly target hemojuvelin (*Hjv*) and hemochromatosis gene (*Hfe*), which are the major regulators in maintaining murine systemic iron homeostasis (Castoldi et al., 2011). miR-485-3p and miR-20a could repress iron exporter ferroportin expression via directly targeting its 3′ UTR in HepG2 cells and in lung cancer, respectively (Sangokoya et al., 2013; Babu and Muckenthaler, 2016). Up-regulation of miR-320 declines the abundance of transferrin receptor on the plasma membrane and impairs iron uptake in the lung cell lines A549 (Schaar et al., 2009). Moreover, miR-Let-7d suppresses DMT1 expression at the mRNA and protein levels, and impairs erythroid differentiation due to endosomal iron accumulation (Andolfo et al., 2010). However, the microRNAs targeting intestinal DMT1 remains elusive.

Here, bioinformatics tools were applied to screen and identify microRNAs targeting DMT1, and then these candidate microRNAs were verified *in vitro* and *in vivo*.

## Materials and Methods

### microRNA Targeting DMT1 Prediction and Sequence Analysis

Four different bioinformatic algorithms were used to predict, screen, analyze, and obtain microRNAs potentially involved in the binding with the 3′ UTR of DMT1. The bioinformatic tools are the following: TargetScan (Friedman et al., 2009), PITA (Kertesz et al., 2007), miRanda (John et al., 2004), and miRDB (Wong and Wang, 2015). Using these bioinformatic tools, mature sequences, seeding sequence and binding sites within DMT1 3′ UTR of candidate microRNAs were identified. Predicted microRNAs targeting DMT1 are represented in Supplementary Table S1.

### Plasmids Construction

To test the direct binding of miR-16, miR-195, miR-497, and miR-15b to the target gene DMT1, we performed a luciferase reporter assay. pcDNA3.1(+)-microRNAs overexpression plasmid, pmirGLO luciferase reportor vector containing 1953 bp fragment of the DMT1 3′ UTR (GenBank Accession No. NM_00128440.1) or the mutated microRNAs’ binding sites of DMT1 3′ UTR were synthesized by Zoonbio Biotechnology Co., Ltd. (Nanjing, Jiangsu, China). microRNAs inhibitors were synthesized by Biomics Biotechnologies Co., Ltd. (Nantong, Jiangsu, China) as single-stranded 2’-O-methyl-modified RNA oligonucleotides. The sense sequences are listed in Supplementary Table S2.

### Cell Culture and Luciferase Report Assay

HCT116 cells (1 × 10^5^/well) were seeded into 24-well plates and cultured in RPMI 1640 (SH30809.01B, Hyclone, Logan, UT, United States) containing 100 IU/mL penicillin, 100 IU/mL streptomycin, and 10% fetal bovine serum (A31608-02, Gibco, Carlsbad, CA, United States) at 37°C in 5% CO_2_ incubator. At 24 h after plating, when reaching a 90∼95% confluence, cells were cotransfected with 50 ng of the luciferase reporter gene construct and 1 gμg microRNA overexpression plasmid or 100 nM microRNA inhibitors using lipofectamine 2000 (11668019, Life Technologies Inc., Waltham, MA, United States). After 24 h, the Firefly and Renilla luciferase signals were detected by the dual-luciferase reporter assay system (E1910, Promega, Madison, WI, United States) following the manufacturer’s instructions. Luciferase activity was examined on the GlOMAX^TM^ 96 microplate luminometer (Promega, Madison, WI, United States). Firefly luciferase activity was normalized to Renilla luciferase activity for each transfected well. The same strategy was adopted for DMT1 3′ UTR mutated forms.

To perform Real-time PCR and Western blot analysis, HCT116 cells (1 × 10^6^/well) were seeded into 6-well plates to reach 80∼85% confluence after 24 h and then transiently transfected with 6 μg microRNA overexpression plasmid. After 24 and 48 h transfection, cells were harvested to perform the experiment.

### Animal and Tissue Preparation

Six-week-old male C57BL/6J mice (15∼18 g, purchased from Shanghai Ling Chang Biological Technology Co., Ltd.) were housed in a controlled room with a light/dark cycle of 12 h and an ambient temperature of 22 ± 1°C, and given free access to food and deionized water. The tail vein injection procedure of miR-16 was performed according to previous studies (Gong et al., 2009, 2010; Fan et al., 2017). After adopting them for a week, mice were divided into two groups (*n* = 15 in each group): negative control plasmid group (miR-SC) and miR-16 over-expression group (miR-16). Plasmid transfection was performed as previously described (Gong et al., 2010). Briefly, miR-SC plasmid (20 μg) or miR-16 plasmid (20 μg) in 150 μL Opti-MEM medium (31985-070, Gibco, Carlsbad, CA, United States) was mixed thoroughly with lipofectamine 2000 (25 μL) in 150 μL Opti-MEM medium, and the total 300 μL mixture was incubated at room temperature for 30 min, then injected into the mice by tail vein. This injection was performed at 9:00 am once every 2 days for five times. Body weight and feed intake were recorded to calculate average daily feed intake throughout the feeding period. At the endpoint, all the mice were sacrificed under general anesthesia after overnight fasting. Blood samples were obtained by orbital venous and stored at −20°C for plasma iron analysis. Duodenum and liver samples were rapidly dissected and frozen at −80°C until use. The animal handling and sampling procedures were consistent with the approved protocol of the Animal Ethics Committee of Nanjing Agricultural University.

### Determination of Iron Concentration

Plasma iron level was measured using the automatic biochemical analyzer (7020, Hitachi High-Tech Crop., Tokyo, Japan) according to the instructions of kits (6063-2012, Shino-Test Corporation, Tokyo, Japan). Iron concentrations in liver and diet were detected by atomic absorption spectrometry according to the method as described previously (Li H. et al., 2017).

### RNA Isolation and Quantification of Mature microRNAs

Total cellular and tissue RNA were extracted by TRIzol reagent (15596026, Invitrogen, Carlsbad, CA, United States) following the manufacturer’s instruction. The concentration of the extracted RNA was detected by the NanoDrop 1000 spectrophotometer (Thermo Fisher Scientific, Wilmington, DE, United States). RNA integrity was confirmed using denaturing agarose electrophoresis. Total RNA (6 μg) treated with RQ1 RNase-Free DNase (M6101, Promega, Madison, WI, United States) was polyadenylated using poly (A) polymerase at 37°C for 1 h with a Poly (A) Tailing Kit (AM1350, Applied Biosystems, Waltham, MA, United States). The polyadenylated RNA (2 μg) was reverse transcribed by poly (T) adapter. Real-time PCR was performed in an MX3000P (Stratagene, California, AC, United States) with SYBR Premix Ex Taq^TM^ II (RR820A, Takara, Otsu, Japan) using a microRNA-specific forward primer and a universal poly (T) adapter reverse primer. Exogenous reference was used as a reference gene to normalize the expression of microRNAs. All the sequences of primers, poly (T) adapter and exogenous reference gene for microRNA are shown in Supplementary Table S3.

### Quantitative Real-Time PCR

After RNA isolation and quality authentication, unify the concentration to 500 ng/μL of each sample, then M-MLV (M1701, Promega, Madison, WI, United States) and dN6 random primer (3801, Takara, Otsu, Japan) were used to synthesize cDNA according to manufacturer’s instructions. Each cDNA generated was amplified by quantitative PCR using SYBR Premix Ex Taq^TM^ II kit (RR820A, Takara, Otsu, Japan) in Mx3000P (Stratagene, California, AC, United States). Peptidylprolyl isomerase A (PPIA) was chosen as a reference gene in duodenum and liver. All primer sequences used for qRT-PCR are listed in Supplementary Table S4.

### Protein Extraction and Western Blot Analysis

Cells or tissue samples were lysed in RIPA lysis buffer (Tian P. et al., 2017) added protease inhibitor (P8340, Sigma, St. Louis, MO, United States) for 30 min on ice, and then centrifuged at 12, 000 rpm for 15 min at 4°C. The supernatant was collected and measured to calculate protein concentration using a BCA protein assay kit (23225, Thermo Fisher Scientific, Waltham, PA, United States). After denaturation, 80 μg protein was electrophoresed in a 10% SDS-PAGE, and then transferred onto a nitrocellulose membrane. The membrane was blocked with 5% skimmed milk powder in TBST (Tris buffer with 0.1% Tween, pH 7.6) for 2 h and then incubated at 4°C overnight with primary antibodies: DMT1 (ab55735, Abcam, 1:500) or β-actin (BS6007M, Bioworld, 1:10000). A secondary HRP-conjugated antibody (BS12478, Bioworld, 1:10000) was used to incubate the membrane for 2 h prior to chemiluminescent detection. The signals were determined using a chemiluminescent substrate (ECL) kit (NCI4106, Thermo Fisher Scientific, Waltham, PA, United States). Then, protein intensities were quantified by the VersaDoc MP 4000 system (Bio-Rad, California, CA, United States).

### Statistical Analysis

The data were analyzed using One-Way ANOVA in SPSS Statistics (Version 20.0, SPSS Inc., Chicago, IL, United States) and presented as mean ± SEM. The 2^−ΔΔCt^ method was applied to calculate the real-time PCR result to obtain the expression fold change. Statistical significance was defined as *P* ≤ 0.05.

## Results

### Screening and Identification of Conserved Target Sites for microRNA-16 Family Within the DMT1 3′ UTR

**FIGURE 1:**
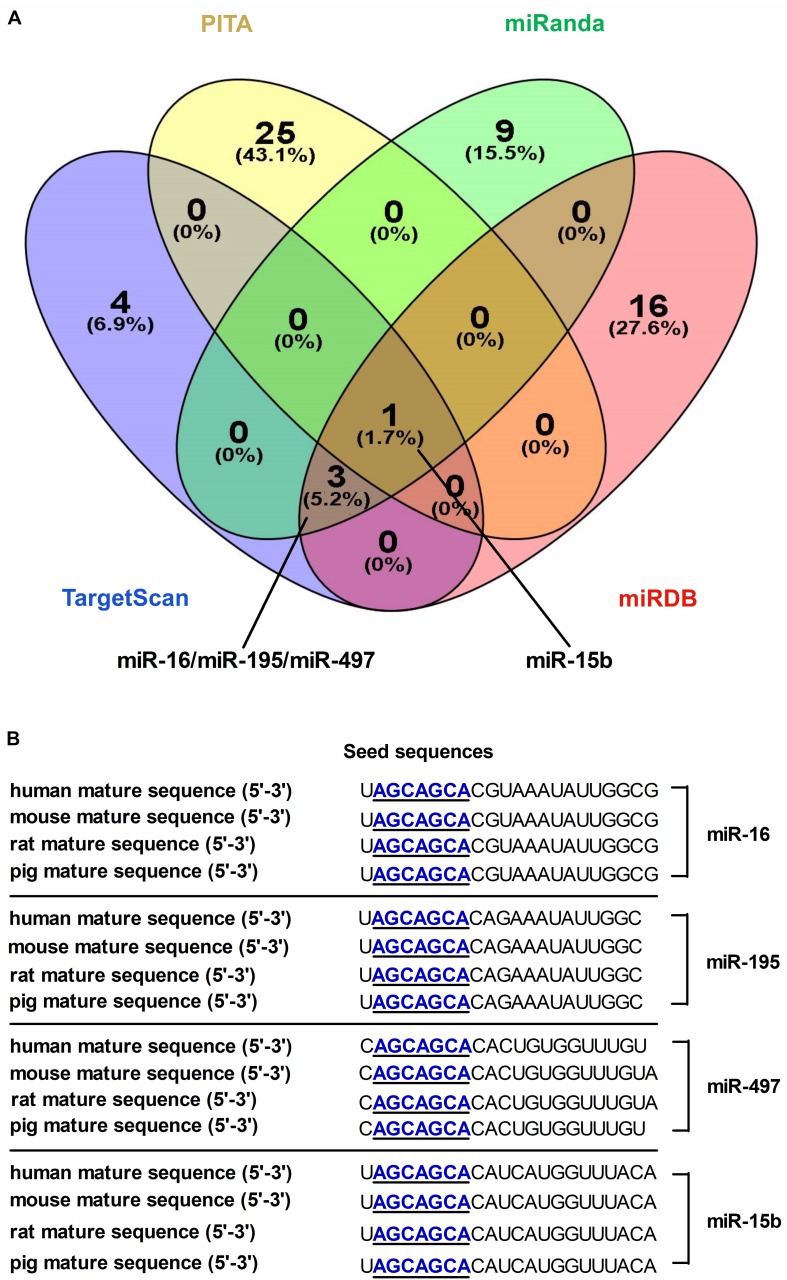
Screening and identification of candidate microRNAs and its seed sequences targeting DMT1 3′ UTR. **(A)** Venn diagram illustrates the number of unique and overlapping microRNAs screened from TargetScan, PITA, miRDB, and miRanda. **(B)** The candidate microRNAs shows highly conserved seed sequences among human, mouse, rat, and pig.

A combination of microRNA target prediction algorithms (TargetScan, PITA, miRDB and miRanda) was used to screen and obtain microRNA-16 family (miR-16, miR-497, miR-15b or miR-195) targeting DMT1 (Figure 1A). Bioinformatic analyses indicated that these microRNAs sequence and its binding sites within the DMT1 3′ UTR sequence are highly conserved among human, mouse, rat, and pig (Figure 1B). Together, these bioinformatic analyses suggested that microRNA-16 family might have highly conserved binding sites within the DMT1 3′UTR.

### microRNA-16 Family Directly Targets DMT1

**FIGURE 2:**
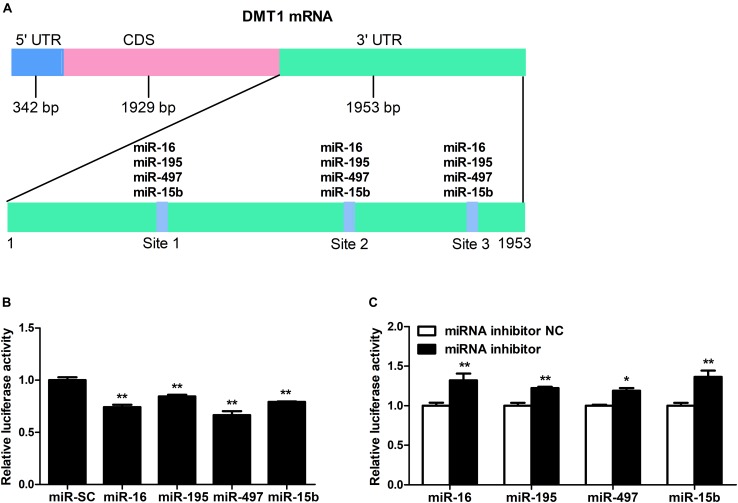
DMT1 is a direct target of candidate microRNA-16 family. **(A)** Schematic description of conserved binding site for microRNA-16 family on the DMT1 3′ UTR region. **(B)** Co-transfection of DMT1 3′ UTR luciferase reporter with microRNA-16 family impairs the relative luciferase activitiy after 24 h. **(C)** Co-transfection of DMT1 3′ UTR luciferase reporter with microRNA-16 family inhibitors enhances the relative luciferase activitiy for 24 h. Values were expressed as means ± SEM, *n* = 6 in each group, ^*^P < 0.05, ^∗∗^*P* < 0.01.

**FIGURE 3:**
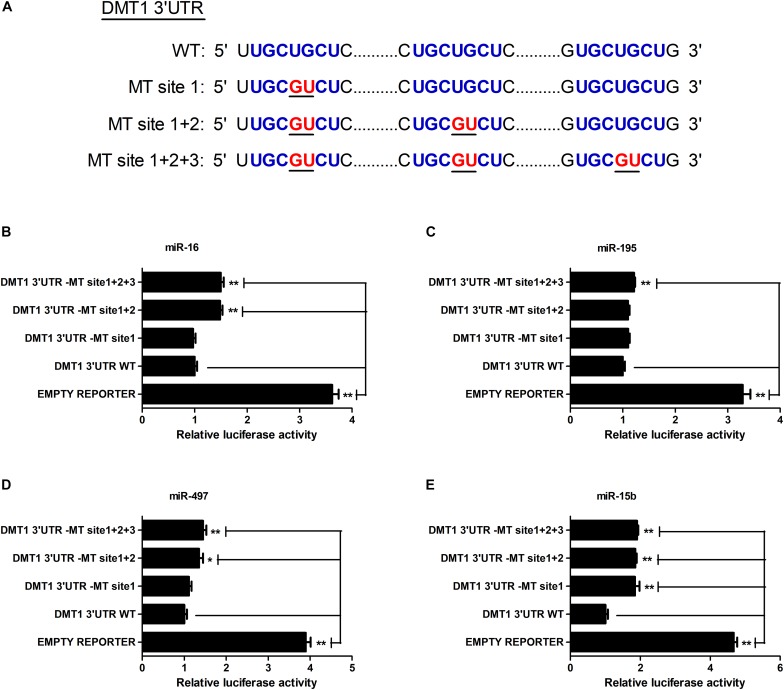
microRNA-16 family directs repression of DMT1 expression validated by the mutant 3′ UTR dual-luciferase reporter. **(A)** Schematic representation of wild type and mutant DMT1 3′ UTR sequences used for the construction of luciferase reporter vectors. **(B–E)** Fold changes of the relative luciferase activitiy in HCT116 cells co-transfected with microRNA-16 family member and mutated DMT1 3′ UTR luciferase reporter/empty luciferase repoter compared to wild type DMT1 3′ UTR luciferase reporter. Values were expressed as means ± SEM, *n* = 6 in each group, ^*^P < 0.05, ^∗∗^*P* < 0.01.

As shown in Figure 2A, we screened the entire 3′ UTR of DMT1 and derived three identical binding sites for microRNA-16 family. Dual-luciferase reporter assay was used for evaluating the relation of microRNA-16 family and its target mRNA (DMT1). Luciferase activity was greatly decreased in response to microRNA-16 family overexpression (*P* < 0.01) (Figure 2B), but increased in HCT116 cells treated with microRNA-16 family inhibitors (*P* < 0.05 or *P* < 0.01) (Figure 2C). To determinate whether the DMT1 3′ UTR was a direct target of microRNA-16 family, we created mutant DMT1 3′ UTR reporters with mutations in predicted binding sites (Figure 3A). Co-transfection experiments implied that microRNA-16 family member (miR-16, miR-497, miR-15b, or miR-195) significantly enhanced luciferase reporter activities in the HCT116 cells treated with microRNA overexpression plasmid and DMT1 mutant reporter containing all the three mutated binding site (*P* < 0.01) (Figures 3B–E). These results further demonstrated that microRNA-16 family directly target DMT1 3′UTR.

### microRNA-16 Family Post-transcriptionally Regulates DMT1 Expression *in vitro*

**FIGURE 4:**
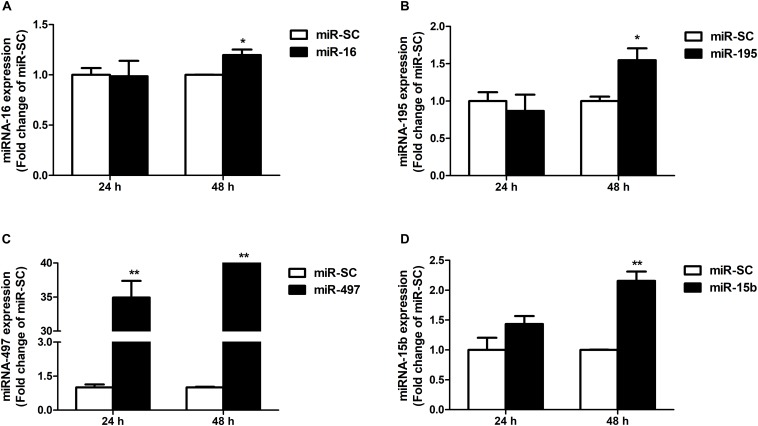
microRNA-16 family overexpression plasmid transfection and its expression in the HCT116 cells. **(A)** qRT-PCR revealed the miR-16 expression in HCT116 cells transfected with moderate corresponding microRNAs for 24 and 48 h. **(B–D)** Expression of miR-195, miR-497, and miR-15b were analyzed at 24 and 48 h after transfection. Values were expressed as means ± SEM, *n* = 6 in each group, ^*^P < 0.05, ^∗∗^*P* < 0.01.

**FIGURE 5:**
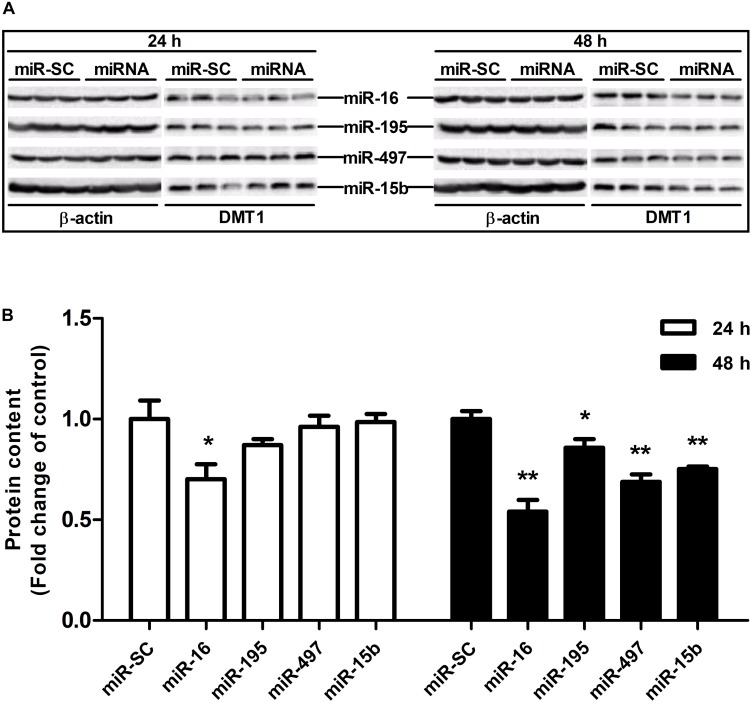
microRNA-16 family regulates endogenous DMT1 expression *in vitro*. **(A)** DMT1 protein expression was analyzed by western blot in HCT116 cells transfected with microRNA-16 family members for 24 and 48 h. **(B)** Densitometric analysis of DMT1 protein expression normalized to β-actin. Values were expressed as means ± SEM, *n* = 6 in each group, ^*^P < 0.05, ^∗∗^*P* < 0.01.

As illustrated in Figures 4A,B,D, overexpression of miR-16, miR-15b or miR-195 significantly raised cellular miR-16 (*P* < 0.05), miR-15b (*P* < 0.01) and miR-195 (*P* < 0.05) expression at 48 h in the HCT116 cells, respectively. miR-497 expression was strongly increased when the cells transfected with miR-497 overexpression plasmid after 24 h and 48 h (*P* < 0.01) (Figure 4C). In addition, overexpression of miR-16 impaired DMT1 protein expression at 24 h (*P* < 0.05), whereas enforced expression of microRNA-16 family members resulted in a big reduction of DMT1 protein expression at 48 h (*P* < 0.05 or *P* < 0.01) (Figures 5A,B). Take together, these results showed that microRNA-16 family directly target endogenous DMT1 and negatively regulate its expression.

### miR-16 Regulates Endogenous Intestinal DMT1 Protein Expression *in vivo*

**FIGURE 6:**
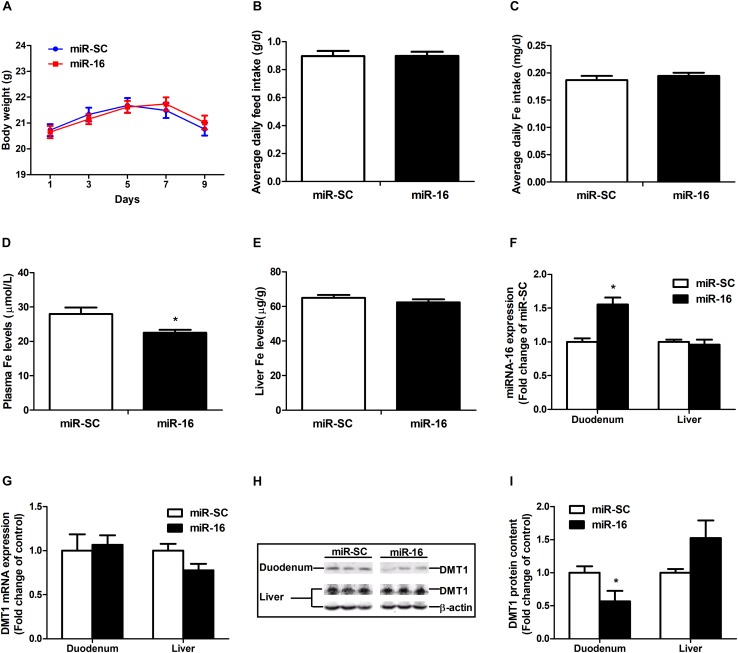
miR-16 regulates endogenous DMT1 expression *in vivo*. **(A)** Body weight was calculated every 2 days. **(B,C)** Average daily feed intake and average daily iron intake throughout the feeding period. **(D)** Plasma iron levels was measured by automatic biochemical analyzer. **(E)** Hepatic iron concentration was detected using atomic absorption spectrometry. **(F,G)** qRT-PCR analysis of miR-16 and DMT1 expression in the duodenum and liver of mice. **(H,I)** Western blot revealed the duodenal and hepatic DMT1 protein expression. Values were expressed as means ± SEM, *n* = 15 in each group for **A–D**, *n* = 6 in each group for **E–I**, ^*^P < 0.05.

miR-16 was selected to verify the function of regulating DMT1. Intravenous tail-vein injection of miR-16 overexpression plasmid did not affect the body weight (Figure 6A), average daily feed intake (Figure 6B), average daily iron intake (Figure 6C), and hepatic iron concentration (Figure 6E) in mice. miR-16 overexpression plasmid injection resulted in a great decrease in plasma iron levels (*P* < 0.05) and a significant increase in duodenal miR-16 expression (*P* < 0.05) (Figures 6D,F). Direct injection of the miR-16 overexpression plasmid had no influence on DMT1 mRNA expression in duodenum and liver, but significantly reduced the abundance of DMT1 protein in the duodenum (*P* < 0.05) (Figures 6G,H,I). In addition, it is acknowledged that equal amounts of duodenal protein samples are loaded onto the gel using coomassie brilliant blue (CBB) staining (Supplementary Figure S1). These results indicated that miR-16 regulates intestinal endogenous DMT1 expression *in vivo*.

## Discussion

microRNAs are now well recognized as important regulators involved in gene expression at post-transcriptional levels. Consideration of different aspects and higher accuracy of prediction algorithms, four microRNA prediction programs (TargetScan, PITA, miRDB, and miRanda) were used to identify candidate microRNAs targeting DMT1. Using this system, we screened and selected four microRNAs (e.g., miR-16, miR-195, miR-497, and miR-15b) targeting DMT1, which belongs to microRNA-16 family based on containing AGCx2 motif in the seed region (Caporali and Emanueli, 2011). microRNA-16 family has been observed to serve as potential oncomiRs or tumor suppressors in various types of cancer (Guo et al., 2013; Liang et al., 2015; Wang et al., 2017; Zhou et al., 2017) and plays an important role in cardiovascular functions in the setting of diabetes (Caporali and Emanueli, 2011). It was proved that the mature sequence and its seed sequence of these four microRNAs were highly conserved between human and rat, mouse, as well as pig based on indification of bioinformatics databases: miRbase (Griffiths-Jones et al., 2006), TargetScan (Friedman et al., 2009), miRanda (John et al., 2004), and miRDB (Wong and Wang, 2015). Nevertheless, the regulation of DMT1 induced by microRNA-16 family needs further verification in multiple important ways.

Generally, microRNAs direct the RNA-induced silencing complex (RISC) to its target mRNA via base pairing between microRNA seed sequence and binding sites of 3′ UTR, and then mediate down-regulation of target gene expression by triggering mRNA degradation or translational repression (Hayes et al., 2014; Iwakawa and Tomari, 2015). The microRNA-mRNA interactions are commonly validated through dual-luciferase reporter assays (Clement et al., 2015; Lo et al., 2017). HCT116 cells is a human colorectal cancer cell line, which is a suitable transfection host^1^, and the proportion of endogenous microRNA-16 family in HCT116 was lower than that in other cells (Cummins et al., 2006; Khan et al., 2009). Moreover, there are many similar studies in HCT116 cells. miR-301a promotes intestinal inflammation and colitis-associated cancer development by inhibiting BTG1 (He et al., 2017). Overexpression of miR-34a may inhibit the proliferation, invasion and metastasis of HCT116 cells (Li C. et al., 2017). Stress-responsive miR-31 is a major modulator of mouse intestinal stem cells during regeneration and tumorigenesis (Tian Y. et al., 2017). miR-29a regulates radiosensitivity in human intestinal cells by targeting PTEN gene (Wang et al., 2016). miR-106b fine tunes ATG16L1 expression and autophagic activity in intestinal epithelial HCT116 cells (Zhai et al., 2013). Therefore, HCT116 cells were used for screening and functional verification of microRNAs targeting intestinal DMT1 *in vitro*.

Co-transfection of dual-luciferase reporter vector containing DMT1 3′ UTR and microRNA-16 family overexpression vector or its inhibitor resulted in opposite effects on luciferase activity, suggesting an association between miR-16 family and DMT1 repression. Meanwhile, mutagenesis of binding sites in the DMT1 3′ UTR blocked repression of the reporter activity. However, as previously reported, there was no linear relationship between the binding sites and the magnitude of repression (Ameres et al., 2007). In light of the earlier discovery, it is reported that the gene expression is influenced by the number and type of microRNA target sites in the affected genes (Khan et al., 2009; Saito and Saetrom, 2012). Denzler et al. (2016) reported that a high-affinity site will contribute more to effective target-site abundance than a low-affinity site (Denzler et al., 2016). Therefore, it may be that the transferred miRNA has different binding ability to different targets sites, which leads to different regulatory effect. The expression of miR-16, miR-195, miR-497 or miR-15b were significantly increased in HCT116 cells exposed to miR-16, miR-195, miR-497, or miR-15b overexpression for 48 h. Moreover, western blot analysis indicated that miR-16, miR-497, and miR-15b were likely to interact with the 3′ UTR of endogenous DMT1, and consequently down-regulated its expression at the posttranscriptional level. These results confirm that microRNA-16 family directly recognizes and binds to the 3′ UTR of DMT1, thereby suppressing DMT1 gene expression *in vitro*.

Intravenous tail-vein injection of microRNAs displays a way of measuring regulatory function to targeting genes *in vivo* (Gong et al., 2010; Pena-Philippides et al., 2016; Lo et al., 2018). In this study, direct injection of the miR-16 overexpression plasmid resulted in a significant increase in miR-16 expression and a great reduction in DMT1 protein expression in the duodenum but not in the liver. Decreasing plasma iron level is closely linked to reduced duodenal DMT1 protein expression, which will lower transport efficiency of ferrous iron from the intestinal lumen into enterocytes (Oates et al., 2000). microRNA is thought to regulate expression of approximately 100∼200 target genes, while the high-level expression of target genes reduces the miRNA’s regulatory effect (Arvey et al., 2010). It is reported that the transfected exogenous miRNAs and endogenous miRNAs competed for available RISC binding, which resulted in dysregulation of target genes, even upregulation of corresponding mRNAs and proteins (Khan et al., 2009; Nagata et al., 2013). In addition, it was reported that long non-coding RNAs (lncRNAs) could function as competing for endogenous RNAs (ceRNAs) through competing for miRNA binding, and thereby regulate each other (Salmena et al., 2011; Thomson and Dinger, 2016). In recent years, a numbers of lncRNAs [LncRNA uc.372 (Guo et al., 2018), SNHG16 (Wang et al., 2018), SNHG1 (Zhang et al., 2016), or MALAT1 (Liu et al., 2018)] were observed to negatively regulate miR-16 family member expression and played a role in pathogenesis of cancers. Moreover, previous studies indicated that the above lncRNAs are highly expressed in the liver and HepG2 cells (Quek et al., 2015; Zhang et al., 2016; Guo et al., 2018; Liu et al., 2018). miRNA’s target dilution and competing endogenous RNAs may play a role in disturbed hepatic miR-16 and DMT1 expression in mice.

In summary, the present data indicate that the miR-16 family members (miR-16, miR-195, miR-497, and miR-15b) are identified to regulate intestinal DMT1 expression *in vitro* and *in vivo*, which may provide a potential target for intestinal iron absorption.

## Ethics Statement

The animal handling and sampling procedures were consistent with the approved protocol of the Animal Ethics Committee of Nanjing Agricultural University.

## Author Contributions

RZ, YN, and WM conceived the project. SJ carried out most of the experiments. SG assisted in the cell culture and Western blotting. HL conducted the animal experiment. WM wrote the manuscript. All authors approved the final version of the manuscript.

## Conflict of Interest Statement

The authors declare that the research was conducted in the absence of any commercial or financial relationships that could be construed as a potential conflict of interest.

## Footnotes

1 https://www.atcc.org/products/all/CCL-247.aspx#generalinformation
