# Supplementary material for: Identification and Functional Verification of MicroRNA-16 Family Targeting Intestinal Divalent Metal Transporter 1 (DMT1) in vitro and in vivo
Source: Front Physiol. 2019 Jun 27;10:819. doi: 10.3389/fphys.2019.00819 (PMC6610423; doi:10.3389/fphys.2019.00819)
Supplement: Supplementary file 1 [file Data_Sheet_1.doc]

**Supplementary materials**

Supplementary materials contain Supplementary Tables S1-S4 and Supplementary Figure S1.

**Table S1.** microRNAs targeted DMT1 prediction using TargetScan, PITA, miRDB and miRanda.

| NO. | TargetScan | PITA | miRDB | miRanda |
| --- | --- | --- | --- | --- |
| 1 | **miR-497** | miR-423-5p | miR-4478 | **miR-497** |
| 2 | **miR-195** | miR-637 | miR-3714 | **miR-15b** |
| 3 | **miR-15b** | miR-1183 | miR-651-3p | **miR-16** |
| 4 | **miR-16** | miR-1275 | miR-3168 | **miR-195** |
| 5 | miR-142 | miR-939 | miR-635 | miR-424 |
| 6 | miR-192 | miR-1207-5p | miR-4324 | miR-340 |
| 7 | miR-215 | miR-744 | miR-6774-5p | miR-377 |
| 8 | miR-375 | miR-24 | miR-6509-3p | miR-186 |
| 9 |  | miR-1184 | miR-4419b | miR-206 |
| 10 |  | miR-1236 | miR-181a-2-3p | miR-223 |
| 11 |  | miR-125a-3p | miR-4459 | miR-410 |
| 12 |  | miR-768-5p | miR-148a-5p | miR-200a |
| 13 |  | miR-646 | miR-7853-5p | miR-141 |
| 14 |  | miR-526b | miR-105-5p |  |
| 15 |  | miR-34a | **miR-195** |  |
| 16 |  | miR-940 | **miR-15b** |  |
| 17 |  | miR-595 | **miR-16** |  |
| 18 |  | miR-638 | **miR-497** |  |
| 19 |  | **miR-15b** | miR-6838-5p |  |
| 20 |  | miR-197 | miR-4655-5p |  |
| 21 |  | miR-503 |  |  |
| 22 |  | miR-623 |  |  |
| 23 |  | miR-636 |  |  |
| 24 |  | miR-512-3p |  |  |
| 25 |  | miR-650 |  |  |
| 26 |  | miR-877 |  |  |
| 27 |  |  |  |  |
| 28 |  |  |  |  |
| 29 |  |  |  |  |
| 30 |  |  |  |  |

Note: The microRNAs predicted to target *Zip14* are shown using four prediction tools including TargetScan (Context++ score percentile ≥ 85), PITA (ddG score ≤ -12), miRDB (Target Score ≥ 85) and miRanda (mirSVR score <-0.85). In bold are overlapping microRNAs of the prediction results.

**Table S2.** microRNA inhibitors sequences

| **Names** | **Sequence** | |
| --- | --- | --- |
|  | sense（5'-3'） | antisense（5'-3'） |
| miR-16 inhibitor | uagcagcacguaaauauuggcg | cgccaauauuuacgugcugcua |
| miR-195 inhibitor | uagcagcacagaaauauuggc | gccaauauuucugugcugcua |
| miR-497 inhibitor | cagcagcacacugugguuugu | acaaaccacagugugcugcug |
| miR-15b inhibitor | uagcagcacaucaugguuuaca | uguaaaccaugaugugcugcua |
| microRNA inhibitor NC | ucacaaccuccuagaaagaguaga | ucuacucuuucuaggagguuguga |

**Table S3.** microRNA and the corresponding primer sequences

| Names | Sequence (5’to 3’) | miRbase Accession |
| --- | --- | --- |
| miR-16 | tagcagcacgtaaatattggcg | MIMAT0000069 |
| miR-195 | tagcagcacagaaatattggc | MIMAT0000461 |
| miR-497 | cagcagcacactgtggtttgt | MIMAT0002820 |
| miR-15b | tagcagcacatcatggtttaca | MIMAT0000417 |
| Universal primer | tagagtgagtgtagcgagca | N/A |
| Poly(T) adapter | tagagtgagtgtagcgagcacagaattaatacgactcactatagg(t)16vn | N/A |
| Exogenous reference | gtgacccacgatgtgtattcgc | N/A |

**Table S4.** Nucleotide sequences of specific primers

| Target genes | Sequences (5’ to 3’) | | Products | GenBank No. |
| --- | --- | --- | --- | --- |
| DMT1 | F:tctgggtgctcctcttgg | R:tgggaccttgggatactga | 122 bp | NM_008732.2 |
| PPIA | F:gggttcctcctttcacaga | R:ccatccagccattcagtc | 225 bp | NM_008907.1 |

**Figure S1**

Figure S1: Coomassie brilliant blue (CBB) staining ensures that equal amounts of duodenal protein samples are loaded onto the gel. Three common housekeeping proteins (β-actin, tubulin and GAPDH) are not suitable for use due to their changed expression between two groups (n=6).
